# Assigning Residents of Emergency Medicine to Screen Patients Before Admission: a Strategy to Overcome Overcrowding

**DOI:** 10.5812/kowsar.22517464.2923

**Published:** 2012-01-15

**Authors:** Hamid Reza Javadzadeh, Amir Davoudi, Farnoush Davoudi, Sadrollah Mahmoodi, Mohammad Reza Ghane, Hasan Goodarzi, Mehrdad Faraji

**Affiliations:** 1 Department of Emergency Medicine, Baqiyatallah University of Medical Sciences, Tehran, IR Iran; 2 Department of Community Medicine, Tehran University of Medical Sciences, Tehran, IR Iran

**Keywords:** Emergency Services, Intervention

## Abstract

**Background:**

The overcrowded hospital is an unsafe one. Overcrowding the emergency department (ED) results in increased patient suffering, prolonged waiting time, deteriorating level of service, and on occasion, a worsened medical condition or even death.

**Objectives:**

This study proposes a strategy to overcome ED overcrowding.

**Materials and Methods:**

The proportion of acute area admitted patients to screened patients (A/S), and the proportion of patients who were finally transferred to inpatient wards (W/A) to those admitted in ED acute area were investigated during 6 consecutive months. Emergency medicine residents were assigned to screen patients before ED admission and afterwards.

**Results:**

The average A/S changed from 82.4% to 44.2% (*P* = 0.028), and the average W/A changed from 28.3% to 51.48% (*P* = 0.028) before and after screening patients respectively. The initiative resulted in 97 less patients in the acute area per day.

**Conclusions:**

Decreased number of acute area admitted patients, and increase W/A proportion showed that the initiative was successful in obviating ED overcrowding while provision of care to those most in need was not altered.

## 1. Background

The overcrowded hospital is an unsafe one. Overcrowding in Emergency Departments (ED) is a common problem, which alters patients’ safety, treatment, privacy and patient satisfaction in addition to increasing the risk of staff frustration. Mitigating overcrowding may have positive effects for patients as well as residents’ (1). Various interventions have been examined in each of three domains of input, throughput and output of ED conceptual model (2, 3) to overcome the problem of ED overcrowding. Two well-known input interventions are “Streaming” (e.g. dividing patients into those who would benefit from admission and those who could be treated as outpatients) and assigning physicians in charge of triage. Our hospital ED, is an academic medium-volume tertiary healthcare ED which has a rapid screening zone, where patients are initially evaluated and separated into two main streams: Those who are to be admitted in ED acute area and those who can be managed as outpatients. Patients used to be evaluated by general practitioners in the rapid screen zone. But from October 2010, EM residents trained by the EM department were assigned to screen patients before being admitted to the acute area.

## 2. Objectives

This strategy was implemented both with educational and managerial objectives; to mitigate ED load of patients.

## 3. Materials and Methods

In this cross-sectional study, which covers the time span from April 2010 to the end of March 2011 assessed the effectiveness of assigning EM residents (instead of general practitioners) to screen patients before being admitted to ED. Based on information provided by Hospital Information System, the number of screened patients (S), those admitted to the ED acute area (A) and those who were finally transferred to inpatient wards (W) were used to evaluate: The A/S proportion to indicate the patient load of ED and the W/A proportion to make sure that critical patients were not diverted to other hospitals. The A/S and W/A proportions 6 consecutive months before (April-October 2010) and after the intervention (October 2010- March 2011) were compared. The Wilcoxon signedranks test was used to compare data means before and after the intervention. *P* -value < 0.05 was considered statistically significant.

## 4. Results

**Figure 1.**
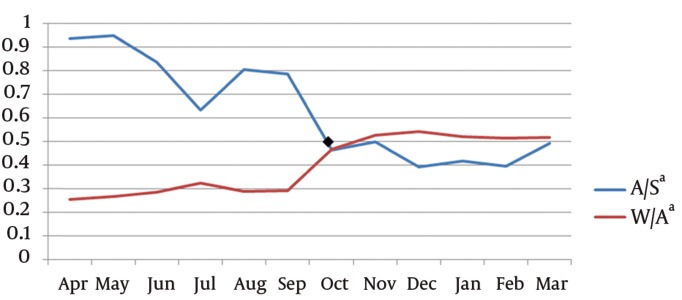
Trends of A/S and W/A proportions ^a^ Abbreviations: A/S, proportion of admitted patients to acute area(A) to screened patients(S); W/A: proportion of patients transferred to impatient wards(W) to admitted patients to acute area(A).

The mean (± SD) number of patients screened before ED admission was 8380.33 (± 658.09) from April to October 2010 and of 8815 (± 887.59) there after (*P* = 0.748). The mean number (± SD) of ED admitted patients before and after the intervention were 6855.33 (± 598.89) and 3738.16 (± 164.1) respectively (*P* = 0.028). This can be translated to 97 less patients per day. The mean number (± SD) of patients who were finally transferred to inpatient wards was 1934.33(± 46.76) before and 1921.00 (± 29.55) after the intervention (*P* = 0.465). The average A/S changed from 82.4% to 44.2% after the intervention (P = 0.028). The average W/A changed from 28.3% to 51.48% in the second half of the study (*P* = 0.028). Figure 1 demonstrates the trend of A/S and W/A proportions over the time period.

## 5. Discussion

According to Ospina and colleges, the total number of ED patients is the second most important indicator of ED overcrowding (4). The study results revealed that ED input has been reduced to less than half, which makes the strategy a successful one. Bittencourt and colleges in a systematic review of interventions to solve overcrowding in hospital emergency services, mentioned patient stream improvement as one of the frequent answers to the problem (5). Kelly (2007) and King (2006) have also demonstrated the effectiveness of such interventions(6, 7). Since EM residents are academically trained to practice triage, they can perform patient streaming more efficiently which in turn reduces the ED acute area admission. Physician triage has proved to be associated with better ED function (8, 9). We believe that EM specialists or residents are the most capable physicians to performed triage, since they have both the necessary knowledge and professional skills. The benefits of assigning such staff to triage has benefits far beyond mitigation of ED overcrowding. While trying to reduce the input of ED, one must make sure that those who need to receive inpatient care are not redirected to other medical facilities instead of being admitted. In the present study we assumed that patients who are finally transferred to inpatient wards are representatives of the afore-mentioned group of patients. The almost constant number of transferral to wards in both arms of the study, and the increased W/A proportion indicates that the strategy has not resulted in patient redirection. Thus, assigning EM residents to screen patients before ED admission can be an effective strategy to overcome ED overcrowding.
